# Editorial: Hypothalamic obesity: Today and future

**DOI:** 10.3389/fendo.2022.1025440

**Published:** 2022-10-03

**Authors:** H.M. van Santen, E. Spinedi, H.L. Muller

**Affiliations:** ^1^ Wilhelmina Children’s Hospital, Utrecht, Netherlands; ^2^ Princess Maxima Center for Pediatric Oncology, Utrecht, Netherlands; ^3^ Centre for Experimental & Applied Endocrinology, CONICET Mar del Plata, Argentina Research Council, La Plata Medical School, Mar del Plata, Argentina; ^4^ Department of Pediatrics and Pediatric Hematology/Oncology, University Children's Hospital, Carl von Ossietzky University, Klinikum Oldenburg AöR, Oldenburg, Germany

**Keywords:** hypothalamus, obesity, craniopharyngioma, diencephalic, resting energy expenditure

Hypothalamic obesity is a rare disease which may have serious impact on patients and their families. The special issue with the Research Topic on “*Hypothalamic Obesity: Today and Future*” provides an update on current concepts on etiology, diagnostics, treatment, and long-term follow up of hypothalamic obesity.

Hypothalamic obesity should be recognized as part of the hypothalamic syndrome (1), consisting of further clinical manifestations such as hyperphagia, sleep disturbances, decreased energy expenditure, hyperinsulinemia, hypopituitarism, psychosocial disorders, memory impairment, attention deficit, reduced impulse control as well as increased risk of cardiovascular and metabolic disorders and reduced quality of life. In addition, many of these patients have visual dysfunction due to either congenital or acquired damage to the optic nerves.

The hypothalamic syndrome is a rare disorder (its epidemiology is not well known because incidence and prevalence are related to very rare underlying diseases caused by disease- and/or treatment-related injury to the hypothalamus), most commonly associated with rare, non-cancerous parasellar masses, such as craniopharyngiomas, germ cell tumours, gliomas, cysts of Rathke’s pouch and Langerhans cell histiocytosis, as well as with genetic neurodevelopmental syndromes, such as Prader–Willi syndrome and septo-optic dysplasia, or disorders with unkown etiology such as the Rapid-onset Obesity with Hypoventilation, Hypothalamic, Autonomic dysregulation, and Neural Endocrine Tumour (ROHHADNET)- syndrome (1).

The diagnosis of hypothalamic syndrome is extremely difficult because of its heterogeneous clinical presentation and the lack of specific markers, and no etiological treatment can be provided due to limited knowledge about its pathological mechanism. Accordingly, intensification of research on diagnostic criteria and treatment of hypothalamic syndrome and especially hypothalamic obesity is warranted.

Of all signs and symptoms that can be encoutered in patients with hypothalamic dysfunction, hypothalamic obesity is one of the most important problems. Hypothalamic obesity not only has huge impact on the patient’s and families social life, and psychological state of the patient (negative self esteem, depression and isolation), but may also have detrimental effects on the cardio-vascular and respiratory system. For this reason, in this special issue, we have collected high level papers on the many different aspects of hypothalamic obesity (Abawi et al.; Amin et al.; Craven et al.; Cvijetic et al.; Dimitri; Muriga et al.; van Roessel et al.; van Schaik et al.).

We hope that the papers published in this Research Topic all contribute to the understanding of hypothalamic obesity and that they may contribute to the development of new approaches in the nearby future to help patients for which hypothalamic obesity has huge impact on daily life.

## Author contributions

All authors listed have made a substantial, direct, and intellectual contribution to the work and approved it for publication.

## Conflict of interest

The authors declare that the research was conducted in the absence of any commercial or financial relationships that could be construed as a potential conflict of interest.

## Publisher’s note

All claims expressed in this article are solely those of the authors and do not necessarily represent those of their affiliated organizations, or those of the publisher, the editors and the reviewers. Any product that may be evaluated in this article, or claim that may be made by its manufacturer, is not guaranteed or endorsed by the publisher.
